# The role of probiotics on the roadmap to a healthy microbiota: a symposium report

**DOI:** 10.1017/gmb.2020.2

**Published:** 2020-08-26

**Authors:** Stacey Lockyer, Marisol Aguirre, Louise Durrant, Bruno Pot, Kaori Suzuki

**Affiliations:** 1 British Nutrition Foundation, London, United Kingdom; 2 Yakult Europe B.V., Almere, The Netherlands; 3 Yakult UK Limited, South Ruislip, United Kingdom

**Keywords:** Probiotics, *L. casei* Shirota, health, gut microbiota

## Abstract

The ninth International Yakult Symposium was held in Ghent, Belgium in April 2018. Keynote lectures were from Professor Wijmenga on using biobanks to understand the relationship between the gut microbiota and health; and Professor Hill on phage–probiotic interactions. Session one included talks from Professor Plӧsch on epigenetic programming by nutritional and environmental factors; Professor Wilmes on the use of “omics” methodologies in microbiome research and Professor Rescigno on the gut vascular barrier. Session two explored the evidence behind *Lactobacillus casei* Shirota with Dr Nanno explaining the plasticity in immunomodulation that enables the strain to balance immune functions; Dr Macnaughtan outlining its potential therapeutic use in cirrhosis and Professor Nishida detailing effects in subjects under stress. The third session saw Professor Marchesi describing that both the host genes and the gut microbiota can play a role in cancer; Professor Bergheim highlighting crosstalk between the gut and the liver and Professor Cani describing the relationship between the gut microbiota and the endocrine system. The final session explored probiotic mechanisms, with Professor Lebeer dissecting the challenges in conducting mechanistic studies; Professor Wehkamp describing the mucosal defence system and Professor Van de Wiele detailing methods for modelling the gut microbiota *in vitro.*

## Introduction

The ninth International Yakult Symposium, entitled “The Role of Probiotics on the Roadmap to a Healthy Microbiota” was held on 19th–20th April 2018 in Ghent, Belgium. This report summarises the talks given by a panel of international experts, who covered different aspects of microbiology, systems biology, immunology, genetics and research methodologies as well as health conditions such as metabolic and liver disease, cancer and stress.

## You and your microbes and the use of population biobanks to understand their intricate relationships

Professor Cisca Wijmenga (University of Groningen, The Netherlands) set the scene for the meeting by outlining the complex ecosystem that is the gut microbiota, comprising bacteria, fungi and viruses. There are hundreds of different bacterial species in the gut (Wallace et al., 2011) and their DNA can now be studied via metagenomic sequencing, currently used to identify which bacterial strains are present, what kind of genes they harbour and their functional pathways (Chen et al., 2018).

The microbiota is extremely dynamic during the course of life. For example, the composition of the microbiota matures from birth towards an adult-like composition in the first 3 years of life and the specific bacteria that colonise the human body will be dependent on various environmental exposures (Chen et al., 2018). Serious perturbations (eg. antibiotic use) that occur in this community both during early life or later can lead to dysbiosis (ie. variation in composition, such as decreased diversity). However, the normal variation present in the gut microbiota, which factors can lead to dysbiosis and how the balance of the gut microbiota can be restored during dysbiosis is not yet fully understood. Dysbiosis has been implicated in diseases that manifest both within and outside the gut including autism, asthma, liver disease, skin disorders, type 2 diabetes, obesity and inflammation (Chen et al., 2018), but these relationships have mostly been reported from observational data and so it is not known whether dysbiosis is causal or occurs as a result of such diseases. If indeed there is causality, knowing which exact strains/species of bacteria contribute to disease, at what time and through which mechanisms, and how the gut microbiota can be modified to treat or prevent disease would be extremely useful, particularly in relation to chronic diseases due to the high proportion of individuals experiencing these during their lifetime.

Population-based cohort studies are useful in studying the gut microbiota and their collective set of genes (ie. microbiome) because they tend to be large in size and the data are collected in a standardised way, therefore interactions between different layers of data (eg. genetic, environmental, behavioural, social) can be examined to try to untangle relationships (Wijmenga and Zhernakova, 2018). Many different phenotypes can be described using “omics” data. Of particular use are longitudinal data which capture people before they have a disease, during the disease and afterwards. More than 160,000 people of the northern region of the Netherlands aged from 8 to 100 years have contributed to the LifeLines cohort study, a longitudinal biobank which began in 2005. Blood, urine, faeces and hair samples, plus a large variety of other data such as heart and lung function, lifestyle questionnaires and dietary information have been collected from the participants. More detailed information is held for a subset of ~1,500 people including multiomics data such as genotypes, transcriptomes, DNA methylation status, blood lipids, untargeted metabolites, proteins and characterisation of the gut microbiome using both 16S rRNA gene sequencing and metagenomics. This cohort is known as LifeLines DEEP (Tigchelaar et al., 2015) and one of the aims of this project is to identify factors that are associated with the composition of the gut microbiota. Two hundred and seven different factors such as blood lipids, diseases, medication, diet and host genetics have been examined. Simple profiling of faecal samples from 1,135 subjects demonstrates the high variability in the gut microbiota between individuals (Zhernakova et al., 2016), with less diversity considered as being less healthy for the host. What is clear is that the gut microbiota is personal and unique for every person. At present, however, what is meant by a “healthy” microbiota is not known because there is no uniform scoring system available. Overall, more than 100 factors have been estimated to contribute to microbiota variation or perturbation (Zhernakova et al., 2016). Strong correlations were observed with blood lipids [eg. levels of high-density lipoproteins and triglycerides were more strongly associated with the microbiota than with host genes (studied by metagenomics)], medication, smoking, diet, body mass index, stool consistency and many conditions including anaemia, irritable bowel syndrome (IBS) and cardiovascular events. Stool consistency (measured by the Bristol Stool Form Scale) and frequency of bowel movements had a strong association with the composition of the gut microbiota in this cohort (Falony et al., 2016). For example, the abundance of Firmicutes tended to be higher (and simultaneously Archaea tended to be lower) in individuals with more frequent bowel movements. However, it is worth mentioning that with respect to this particular association, the literature shows no clear consensus.

Data obtained in the same cohort suggest a striking effect of medication on the gut. In particular, commonly used drugs such as statins and antidepressants have been associated with lower diversity of the gut microbiota (Zhernakova et al., 2016). Furthermore, proton pump inhibitors (PPIs) have been linked with increased risk of *Clostridioides difficile* and *Campylobacter* infections (Imhann et al., 2017). In the Lifelines DEEP cohort, PPI use was associated with a 20% difference in the taxa that were typically present (Imhann et al., 2016). Bacteria that are normally only present in the oral cavity were now found in the gut of these individuals. This was presumably because the pH of the stomach is increased by the PPIs, reducing the acidic barrier between the oral cavity and the gut, hence increasing the risk of enteric infections. Diet was also found to be a major indicator of gut diversity among the subjects and some dietary components could be considered unfavourable as they were associated with decreased diversity (Zhernakova et al., 2016). These include whole fat milk, beer, fizzy drinks, total intake of carbohydrates and high calorie diets; whereas fruits, vegetables, coffee, tea, red wine, buttermilk, cheese and yoghurt were associated with increased diversity. *Streptococcus thermophilus*, a traditional starter culture for the elaboration of yogurt, was highly abundant in the stools of daily consumers.

Professor Wijmenga finished by describing forthcoming extensions of her research. The LifeLines-NEXT cohort is expected to comprise 1,500 newborns that are the offspring of LifeLines participants. Data will be collected at multiple time points during the first year of life, hoping to shed light on the role of the virome 2016 in shaping the gut microbiome in its early development stage. Another project is the Netherlands Organ on a Chip Initiative (Netherlands Organ-on-Chip Initiative, 2018), a 10-year project centred around the recreation of organs-on-chips to look at, eg. the interaction between the gut microbiota, the gut epithelium or the liver using 3D microfluidics. The methodology used in this project aims to provide an environment that is more physiological than other *in vitro* methods to study tissue function in health and disease.

## The interaction of epigenetics and microbiota development

Dr Torsten Plösch (The University Medical Center Groningen, The Netherlands) talked about the interaction of epigenetics and microbiota development, focusing on the long-term effects of nutrition in early life.

Dr Plösch cited the work of Dr David Barker who was one of the first to describe the relationship between undernutrition *in utero* and the proneness to diseases later in adult life. Barker was interested in why there were higher rates of cardiovascular disease (CVD) in some areas of the UK than others, and hypothesised that this was linked to early nutrition (Barker and Osmond, 1986). By analysing medical records, he found a correlation between birthweight and CVD risk with both low and high birthweight predicting a higher chance of developing CVD in later life. A similar U-shaped curve was found for type 2 diabetes (Barker et al., 1993). Studies looking into the Dutch Famine of 1944 demonstrated that starvation during pregnancy has detrimental effects on the long-term health of the offspring (Painter et al., 2005).

These observations are partially explained by epigenetics. Epigenetics can be illustrated by caterpillars and butterflies which have the same DNA but differently expressed according to the life stage, or by organ-specific differentiation of human cells which contain identical DNA (eg. skin cells, neurons, liver cells). Epigenetics can broadly be described as any modification that turns the expression of genes on and off, encompassing chromatin and DNA modifications and other transcription regulators (eg. DNA methylation at the CpG sites or acetylation of histones) (Greally, 2018).

Epigenetic status is influenced early on in development and then remains relatively stable. Dr Plösch hypothesises that the early environment leads to epigenomic modifications of key regulators of metabolism which influence lipid metabolism and CVD risk later in life. In a study looking at protein restriction, mice were fed either a normal diet (18% protein) or a low protein diet (9%) before becoming pregnant (van Straten et al., 2010 2009). Epigenetic characterisation of foetal livers revealed that 204 gene promoters were differentially methylated between the two groups. One of the most interesting genes was the liver X-receptor (LXR) which regulates lipid homeostasis. Under normal circumstances LXR is activated by oxysterols, cholesterol side products that stimulate adenosine triphosphate-binding cassette (ABC) transporters to pump cholesterol out of the cell and increase lipogenesis, thereby esterifying cholesterol and protecting the cell against cholesterol build up (Steffensen and Gustafsson, 2004). Upon protein restriction, the LXR promoter is hypermethylated; therefore, expression goes down, as does the expression of target genes such as ABCA1 and fatty acid synthase genes 2009. This and the follow-up control experiments (van Straten et al., 2010) provide evidence that diet can induce changes in gene expression via epigenetic programming. Further experiments in mice suggest that epigenetic marks can persist beyond the immediate offspring. After 50% calorie restriction during gestation, methylation of LXR in the liver (but not all organs) of second-generation mice was still altered and associated with abnormal lipid metabolism (Martínez et al., 2014).

Several animal and human studies have indicated that other major regulators of metabolism can be altered in an epigenetic way (eg. peroxisome proliferator-activated receptors, retinoid X receptor, glucocorticoid receptor, ABC transporters, pro-opiomelanocortin and insulin-like growth factor 2 receptor) and it is likely that a combination of these can be affected by the maternal diet, making the offspring more susceptible to the development of chronic disease (Jiménez-Chillarón et al., 2012). One of the proposed mechanisms is the availability of methyl groups for DNA methylation via the one carbon cycle, components of which are derived from the diet (eg. choline, betaine) or generated by gut bacteria (eg. vitamins B1, B2, B6, B9 and B12). It has also been discovered that docosahexaenoic acid has demethylating activity and α-ketoglutarate can regulate DNA methylation. Acetylation of histones is also influenced by diet and by gut bacterial metabolites. For example, butyrate [and other short chain fatty acids (SCFA), albeit to a lesser extent] along with phenolic compounds, organic acids and vitamins B1 and B3, can potently block deacetylation leading to more active chromatin; while the production of sulphur compounds and vitamins B1, B5 and B7 cause other histone modifications (Mischke and Plösch, 2016).

As such, the correlations between the composition of the gut microbiota, diet and health or disease (eg. obesity) observed in both mice (Parks et al., 2013; Serino et al., 2012) and humans (David et al., 2013; Le Chatelier et al., 2013; Ridaura et al., 2013) could be due to epigenetic modifications (Mischke and Plösch, 2013). Causality, however, remains to be elucidated. For example, breastfed babies tend to have more bifidobacteria and ruminococci than formula fed babies (that have more streptococci and clostridia). Since folate (vitamin B9) can be produced by some bacteria including bifidobacteria, theoretically, breastfeeding stimulates folate production which could enhance DNA methylation; whereas formula feeding tends to increase butyrate, an important inhibitor of histone deacetylase (Mischke and Plösch, 2013). Therefore, changing the gut microbiota either through diet or drugs could alter the long-term physiology of the host by changing the availability of substrates for bacteria involved in DNA methylation (Mischke and Plösch, 2016).

In summary, nutrition during the first 1,000 days of life (including the gestational period) is especially important for epigenetic modifications and the early gut microbiota likely plays a significant role in this process. However, there are still many questions left to be answered: are there critical windows of susceptibility during development? Is the bacterial production of metabolites enough to induce epigenetic changes? Which tissues are most vulnerable to these modifications? These questions will likely be answered by ongoing and future research.

## Systems ecology of microbiome-human interactions: identifying which functions are key

Professor Paul Wilmes (University of Luxembourg, Luxembourg) described the integrated multiomics approach that his team has developed to study microbial ecosystems.

High-throughput “omic” technologies unravel the complexity of the microbiome by providing high resolution qualitative and quantitative datasets on the genes (the metagenome), transcripts (the meta-transcriptome), proteins (the metaproteome) and metabolites (the metabolome) present in microbial communities at specific points in space and time (Heintz-Buschart and Wilmes, 2018). These data can be integrated in a “multiomics” approach to reconstruct networks of constituent populations, deduce the interactions within the system (eg. gene regulatory networks, metabolic networks) and provide mechanistic insight into both biotic and abiotic factors shaping microbial communities (Muller et al., 2013).

Meaningful integration and analysis demand systematically generated data, which in turn requires robust sampling, sample preservation and biomolecular isolation methodologies (Roume et al., 2013). Professor Wilmes and his team developed a methodological flow for the sequential extraction and purification of all known biomolecular fractions from single unique samples (Kitano, 2001; Roume et al., 2012). This framework provides a standardised workflow to resolve keystone genes encoding key functionalities that have an effect on the entire ecosystem, keystone species that contribute to the stability of the microbial community, niche characteristics, and specialist and generalist lifestyle strategies. From this information hypotheses can be made around which species govern the success of the system and why or how proteins link to specific populations (if possible at a strain level). In addition, associations between biomolecules that may be indicative of previously unknown metabolic processes linked to specific community members can be uncovered (Roume et al., 2015). This knowledge should allow researchers to conduct molecular investigations on microbial communities with different compositions, compare the molecular profiles obtained and discover functional microbiota-specific biomolecular signatures, eg. substrates linked to different community members. The data can also be used to reconstruct metabolic networks, enabling, eg. to discern between “generalists” and “specialists”. The former are able to thrive in a wide variety of environmental conditions, can use a variety of different resources and are favoured during environmental instability. The latter grow well only in a narrow range of environmental conditions, have a limited substrate usage and are likely to be favoured during environmental stability (Muller et al., 2014).

Perturbations, however, can occur in a microbial community, and analysing time points before and after such events can reveal how a disturbance can affect the whole network. In the context of the human gut, perturbations can be caused by drugs, especially antibiotics, or by dramatic changes in diet (Greenhalgh et al., 2016). Alterations of certain keystone species may have consequences for the whole system. For example, if a keystone species is eradicated, the network is “reset” by opening a significant niche for other organisms which may be opportunistic pathogens or beneficial microorganisms. This phenomenon is important for probiotics in terms of how these niches can be opened, offering a long-term foothold for these organisms within the gut (Quigley, 2010).

In such a setting, integrated multiomics have been used to study links between the gut microbiota and health and disease, eg. the understanding of the relationship between alterations in the microbiota and the functional consequences for type 1 diabetes (T1D). Integrated multiomic analyses were performed in 20 individuals from four Luxembourgian families of at least two generations with at least two cases of T1D (Heintz-Buschart et al., 2016). Faecal samples and dietary data were collected over a period of 2–4 months. Gut microbiota were compared to those without the disease in search for the most discriminative phenotype that could possibly link specific symptoms of the disease with the functions of certain microbial taxa. Individuality and family resemblance were observed at all the omic levels but there was no taxonomical signature for T1D. Rather, the faecal proteome was found to be more distinctive between healthy and diseased individuals. Enzymes secreted via the exocrine pancreas (such as alpha-amylases) involved in starch metabolism were reduced in the stools of individuals with T1D and different taxa (not necessarily abundant) were involved in this function. This observation was also correlated with reduced expression of key microbial enzymes involved in thiamine biosynthesis and glycolysis (Heintz-Buschart et al., 2016).

Professor Wilmes’ group is also interested in changes following the early colonisation and succession of the microbiota during the first year of life from a functional point of view. They found that lipopolysaccharide (LPS) biosynthesis in the gut of infants born by caesarean section is different from those born through vaginal delivery and between infants who were smaller than average versus normal birthweight (Wampach et al., 2017). LPS extracted from vaginally delivered babies was found to be more immunostimulatory in early life than LPS extracted from babies delivered by caesarean section (Wampach et al., 2018). Integrated multiomics allowed linking of these functions to individual strains that are transferred from mother to neonate during vaginal delivery. The long-term functional repercussions of this for infants can be investigated using gut-on-a-chip methodologies, such as the modular microfluidics-based human–microbial coculture (HuMiX) model (Shah et al., 2016). Using these types of tools, multiomics approaches will offer exciting prospects for studying and unravelling interactions during probiotic administration.

In his conclusion, Professor Wilmes emphasised that microbiota *functionality* is more important than *composition*, and in this context, probiotics should be thought of as “bags of functions”, rather than a specific (combination of) species.

## The gut barrier and the immune system in health and disease

### The barrier

The coexistence between microbes and host demands a certain equilibrium which implies that: (i) the host must find the right balance to retain the microorganisms without the exacerbation of inflammatory tone and (ii) the microbial community does not represent a health threat in terms of composition and metabolic activity (Rescigno, 2017). Professor Maria Rescigno (Humanitas University, Italy) described the existence of a vascular barrier in the gut that protects the organism against the development of inflammation and the translocation of bacteria or their metabolic products into the liver and other organs.

Despite the crucial role of the microbiota in supporting the proper functioning of the body, most of the bacteria do not interact directly with the host. In the large intestine, microbes are spatially separated from the epithelium by a two-layered mucus. The outer layer serves as a unique microbial niche for distinct communities, whereas the inner layer constitutes a barrier in which glycoproteins organised in tight net-like structures and antimicrobial peptides reduce the presence of bacteria (Hooper and Macpherson, 2010). If microbes were to cross these layers, they would encounter the gut epithelium, a highly regulated semipermeable cellular barrier. However, mucosa-based protection against bacteria goes beyond the establishment of physical barriers. The mucosal immune system, an interconnected network of inductive sites [eg. mesenteric lymph nodes (mLNs)] and effector sites (eg. lamina propria), is able to selectively restrict the trafficking of exogenous substances and microorganisms into the blood (Spadoni et al., 2016). In a pathologic state, if bacteria translocate through the mucus layers and the epithelium, they are captured by the mLNs via the lymphatic vessels localised in the lamina propria and only microbes capable of systemic dissemination will reach the liver via the portal vein (Spadoni et al., 2017).

Anatomically, blood vessels are much closer to the epithelium as compared to the lymphatic vessels located deeper down (Bernier-Latmani et al., 2015; Cifarelli and Eichmann, 2019). Judging from this distribution, it is expected that bacteria would easily access the blood vessels. Yet, this is not the case most of the time. This suggests the existence of a differential trespassing of bacteria, but the precise mechanism behind the exclusion of gut microbes from the blood circulation is not well understood to date.

Professor Rescigno’s research group postulated and demonstrated the existence of a gut vascular barrier (GVB) similar to the blood–brain barrier (BBB). While these two barriers share some structural and functional properties, as well as the involvement of the Wnt/β-catenin signalling pathway in the control of the translocation of antigens, molecules, bacteria and cells into the blood stream, they differ in their permissiveness. The BBB avoids the uncontrolled migration of any substance in order to not only protect the brain parenchyma but also the central nervous system, whereas the GVB remains permeable to nutrients, facilitating its characteristic absorption function (Spadoni et al., 2016). For this reason, endothelial cells of the BBB do not have fenestrations whilst those of the GVB do.

Professor Rescigno’s group demonstrated the existence of a GVB in mice by analysing the permissiveness of the gut vasculature using the dye fluorescein isothiocyanate (FITC)–dextran of different molecular sizes and examining the expression of plasmalemma vesicle-associated protein-1 (PV1), an endothelial cell-specific protein that makes up the diaphragms of the fenestrae of the capillaries in intestinal villi. Next, they orally administered *Salmonella typhimurium*, a pathogen that disseminates systemically in mice, to investigate whether damage of these structures would allow indiscriminate transit through the endothelium. This resulted in increased FITC-dextran leakage into the intestine, up-regulation of PV1 and liver damage, suggesting that the bacteria damaged the GVB allowing its dissemination via the portal blood to the liver (Spadoni et al., 2015). Further exploration proved that this was caused by a protein secreted by *Salmonella* that interferes with the Wnt/β-catenin signalling pathway. No PV1 staining was observed when gut sections of healthy mice and humans were examined, providing evidence that a GVB is intact (Spadoni et al., 2015). Interestingly, *Salmonella* was also detected in Peyer’s patches, mLNs and the spleen, indicating that the systemic spreading occurred by two different routes, namely the lymphatics and the portal vein.

The studies with *S. typhimurium* also highlighted the concept of the “leaky gut” and the existence of a gut-liver axis, illustrating the cascade of effects originally induced by a gut microbiota dysbiosis (ie. bloom of pathogens). An unbalanced microbiota can result in epithelial and endothelial damage, meaning that bacterial products can translocate to the liver, leading to chronic inflammation and ultimately to metabolic diseases such as cirrhosis (Wiest et al., 2014). The GVB tightly controls this axis and modifications at this barrier may be responsible for the development of liver damage.

Professor Rescigno subsequently demonstrated the existence of a GVB in the human gut and investigated whether the disruption of this barrier could be involved in the development of coeliac disease pathology. Intestinal biopsies analysed from these patients, who additionally had high transaminases levels, revealed high PV1 expression when compared to patients with normal transaminases (Spadoni et al., 2015). Increased transaminases levels are indicators of GVB modifications, presumably leading to liver damage.

In conclusion, there are two cellular barriers in the gut: the epithelium and the vascular endothelium. Although independent, they act in concert to protect not only the intestine but the whole organism from undesirable molecules and microorganisms. Disturbance of these barriers may underlie the pathophysiology of different health conditions.

### The immune system

Professor Jan Wehkamp (University of Tübingen, Germany) talked about the relationships between probiotic bacteria and the host and their application in the development of therapies in combination with immunological or pharmaceutical agents for inflammatory and metabolic diseases.

He started his presentation by emphasising the importance of the intestinal mucosal defence system that keeps us free from inflammatory responses or infections, despite the fact that only one layer of cells separates the rest of our body from the trillions of bacteria present in the gut. As mentioned previously, the mucus layer is a reservoir for antimicrobial peptides (AMPs), eg. defensins and cathelicidins, among others. These peptides mainly act by disrupting the cell membrane of bacteria but can have other mechanisms of action. For example, α-defensin 6 can form “nanonets” which physically prevent bacterial translocation (Raschig et al., 2017). Interestingly, the function of some of the AMPs can vary depending on local environment, eg. human β-defensin (hBD)-1 is ubiquitously expressed on all cell surfaces and while it is not very active against bacteria in aerobic conditions, it exerts a potent antimicrobial effect in the anaerobic environment of the gut due to the reduction of disulphide bonds (Lehrer, 2011; Schroeder et al., 2011).

Some AMPs are secreted by Paneth cells in the small intestine and transgenic mice studies have demonstrated that the amount of defensins expressed in these cells is linked to the composition of the gut microbiota downstream from the small intestine (Salzman et al., 2009; Wehkamp et al., 2005). The involvement of commensal bacteria in physiological and/or pathological processes has been recognised beyond “infection”, eg. being linked to inflammation and carcinogenesis. For instance, inflammatory bowel disease (IBD), previously considered to be an autoimmune disease, is now thought of as a disease linked to a deficient barrier function. In Crohn’s disease, Paneth cells appear to play a central role especially when the inflammation affects the small intestine (Wehkamp and Stange, 2010). The different mechanisms causing different barrier dysfunctions promote and trigger what can be described as a secondary inflammatory cascade related to the presence and influx of commensal bacteria (Antoni et al., 2014).

More and more clinical treatments for cancer and IBD are combined with modulators of the gut microbiota composition (Sivan et al., 2015). In clinical trials in oncology, the clinical outcome of patients treated with checkpoint inhibitors correlates with gut microbial diversity and improved survival (Gopalakrishnan et al., 2018; Routy et al., 2018). Anti-tumour necrosis factor is widely prescribed for IBD patients and it has been reported that this treatment suppresses interleukin (IL)-22 binding protein, thus activating IL-22 (Pelczar et al., 2016), a strong stimulator of innate immunity at the epithelial level and an up-regulator of the antimicrobial peptide hBD-2 (Wolk et al., 2004). This is a rationale for developing drugs which target the mucosal–microbiota interface.

Intriguingly, many probiotics can produce AMPs themselves next to inducing the endogenous production of human defensins (Sassone-Corsi et al., 2016). Various strains including *Escherichia coli* Nissle, lactobacilli and the bacterial mixture VSL#3, are consistent inducers of hBD-2 in intestinal epithelial cells, both *in vitro* and *in vivo* (Mondel et al., 2009; Schlee et al., 2007, 2008; Wehkamp et al., 2004). *E. coli* Nissle is unique among *E. coli* strains in its potency to induce hBD-2 and this effect is thought to be due to mechanisms involving flagella. However, other probiotic strains, not possessing flagella, can also induce hBD-2 but the reasons for this are not yet known (Wehkamp et al., 2004). Inducing hBD-2 appears to be detrimental to probiotics as their incubation with hBD-2 leads to cell death, although it is not clear why or how this occurs. This seemingly “suicidal” action may in part explain why probiotics need to be consumed daily.

This observation led Professor Wehkamp to study the impact of hBD-2 on the microbiota composition and abundance. He observed that the diversity of the gut microbiota increased in mice that received hBD-2 orally but returned to baseline when the treatment stopped (Koeninger et al., 2018). This result and the fact that hBD-2 improves barrier function, led to the hypothesis that hBD-2 could be useful to treat inflammation in colitis. Further experiments indeed showed that hBD-2 was as effective as steroids in mouse models of IBD (Koeninger et al., 2018). Clearly, this finding may be of value in patients as steroids have serious side effects upon long-term use. hBD-2 has also been tested in models of metabolic syndrome and non-alcoholic fatty liver diseases (NAFLDs), with positive results in relation to fat mass and weight control.

These findings illustrate that the modulation of gut microbiota composition to influence barrier function can work synergistically with drug therapies. Understanding the mechanism, eg. induction of protective factors such as IL-22, is crucial.

Having an intact antimicrobial barrier can protect from inflammation, not only in the gut but more widely, and could therefore play a role in the treatment of certain inflammation-related diseases such as asthma and metabolic syndrome.

## The gut and the liver: two of a kind?

Professor Ina Bergheim (University of Vienna, Austria) further discussed the cross-talk between the gut and the liver, namely in the context of alcoholic and non-alcoholic fatty liver disease. Together with mucus, enterocytes, and endothelia in the intestine, the liver is one of the body’s defence mechanisms against substances derived from food, drugs, exo- and endotoxins, xenobiotics and viral compounds. In this sense, the gut and the liver are “two organs that work in concert in a line of defence”.

Approximately 80–90% of individuals with regular high alcohol consumption develop liver steatosis (fatty liver). While steatosis is reversible by stopping drinking alcohol, the organ damage can advance to steatohepatitis, fibrosis, cirrhosis and eventually to hepatocellular carcinoma in a more limited number of patients (Altamirano and Bataller, 2011).

Alcohol is energy rich, providing 7 kcal/g. When consumed, it is metabolised by alcohol dehydrogenase (or cytochrome P450 2E1 during chronic consumption) to acetaldehyde, producing metabolites that are used to synthesise high energy substrates (eg. fat) and concomitantly generate reactive oxygen species (ROS) (Molina et al., 2014). For many years this was thought to explain the relationship between alcohol consumption, fatty liver and liver damage. However, the identification of elevated endotoxin levels in the blood of patients with alcoholic liver disease (ALD) suggested the involvement of a disrupted gut barrier, associated with the stage of ALD (Parlesak et al., 2000; Purohit et al., 2008). Experiments in animal models demonstrated that even acute exposure to ethanol (60–100 min) increases gut barrier permeability (Rivera et al., 1998). Longer alcohol consumption (ie. 10 days) induced the expression of several toll-like receptors (TLRs) in liver tissues in rodents, indicating that not only bacterial endotoxin, but other toxins and viral compounds reach the liver under these conditions (Gustot et al., 2006).

Preclinical and clinical studies have demonstrated that alcohol also alters the gut microbiota composition (Chen et al., 2011; Mutlu et al., 2012). In a rodent model of alcohol-induced liver injury, the administration of non-absorbed antibiotics decreased alcohol-induced damage as compared to a group that did not receive antibiotics (Adachi et al., 1995). Indeed, germ-free mice that received gut microbiota content from patients with more severe alcoholic hepatitis developed much more severe liver damage (Llopis et al., 2016). In another study, alcohol-fed mice receiving either a faecal transplant from mice resistant to alcohol-induced liver damage or mice fed with a pectin-enriched diet, did not develop steatosis or steatohepatitis, whereas control animals receiving neither treatment did develop liver disease (Ferrere et al., 2017). In patients with ALD, the composition of the small intestinal microbiota changes towards a more faecal type, with elevated endotoxin levels. Moreover, the faecal microbiota has been found to differ between regular drinkers with and without inflammation (Vassallo et al., 2015). Overall, ALD seems to be, at least in part, a disease of the gut, although more trials in humans are needed in this area to confirm this finding and to reveal the molecular mechanisms involved.

The global prevalence of non-alcoholic liver disease (NAFLD) is estimated at 24% (Younossi, 2018); the main risk factors are obesity and insulin resistance, pointing to an effect of poor diet. Animal models suggest that a diet high in fat and fructose leads to overweight/obesity and worsens NAFLD (although the amounts consumed were much higher than those typically consumed by humans) (Sellmann et al., 2015). These effects are associated with a loss of tight junction proteins in the small intestine and with elevated bacterial endotoxin levels in portal plasma. The findings are similar to what is observed in models of ALD, suggesting that the gut is also involved in the development of NAFLD (Sellmann et al., 2015). In fact, mice consuming a “Western style diet” (high in fat, fructose and cholesterol) developed non-alcoholic steatohepatitis even in the absence of overnutrition, as compared to an isocaloric control diet. This observation suggests that the nutritional composition of the diet, rather than simple overnutrition, is a possible cause (Sellmann et al., 2015). Human studies have also shown that patients with different stages of NAFLD tend to have (i) increased endotoxin levels in the periphery (Nier et al., 2017), (ii) augmented gut permeability (Volynets et al., 2012), (iii) loss of tight junction proteins in the intestine (Miele et al., 2009) and (iv) upregulated TLRs in their liver tissue (Kanuri et al., 2015).

Regarding the involvement of the gut microbiota in NAFLD, mice fed drinking water enriched with fructose and given antibiotics had ~50–60% reduced hepatic lipid accumulation and expression of TLRs, as compared to those receiving fructose-enriched drinking water alone (Bergheim et al., 2008; Wagnerberger et al., 2012). Furthermore, faecal microbiota transplants from animals consuming a control diet to animals with high fat diet-induced NAFLD attenuates steatohepatitis, restores tight junctions and lowers bacterial endotoxin significantly (Zhou et al., 2017). In humans, faecal microbiota and associated metabolites differ between children with or without NAFLD and vary with differing disease severity (Del Chierico et al., 2017).

The administration of probiotics in children and adults with NAFLD has improved markers of liver function (eg. alanine amino transferase, gamma-glutamyl transferase) in some studies (Aller et al., 2011; Nabavi et al., 2014; Vajro et al., 2011). However, further studies are needed to examine the possible benefits of probiotics in NAFLD (Sáez-Lara et al., 2016).

In summary, there is abundant evidence that dietary patterns and/or alcohol, the intestinal barrier and the gut microbiota contribute to the pathophysiology of ALD and NAFLD. Better understanding of these constituents is necessary to improve preventive and therapeutic measures.

## The gut and the endocrine system: a peaceful combination?

Professor Patrice D. Cani (UCLouvain, Belgium) began his talk by highlighting the discovery of prebiotics, first described over 20 years ago (Gibson and Roberfroid, 1995). Prebiotics are substrates that are selectively utilised by host microorganisms, conferring a health benefit (Gibson et al., 2017). In early experiments it was observed that rats receiving prebiotics (eg. oligofructose, inulin) had a lower body fat mass but the mechanisms of action behind this were not understood. It was also noted that the rodents were consuming fewer calories (Cani et al., 2004) and that regardless of the disease model (eg. type 2 diabetes model or diet-induced obesity model), prebiotic treatment reduced glycaemia and improved insulin sensitivity (Cani et al., 2005a, 2006, 2007b, 2008; Everard et al., 2014). To understand these findings, researchers focused on the gut–brain interaction taking place while food is consumed. Different experiments have shown that the generation of different signals in the lower part of the gut can circulate and reach the brain. These signals include gut peptides [eg. glucagon-like peptide-1 (GLP-1) and peptide YY (PYY)] secreted by L-cells that have been seen to interact with specific receptors present in the portal vein and gut nerves (Chambers et al., 2015a).

In the brain, the integration of food derived-signals coming from the gut and from orexigenic (appetite-increasing) or anorexigenic (appetite-suppressing) neurons, is balanced. Such a process is key for controlling food intake and energy expenditure (Sohn, 2015). Interestingly, in the aforementioned rodent studies prebiotic intake was found to increase the production of GLP-1 and PYY and decrease the production of ghrelin. Furthermore, the studies showed that the expression of genes encoding proglucagon (a precursor of GLP-1) was increased in the lower part of the gut (Cani et al., 2004, 2005a, 2006, 2007b, 2008, 2009). This effect was reproduced in other experiments using different prebiotics (eg. resistant starch) and could possibly be explained by the activity of microbial metabolites generated from the fermentation of different prebiotic fibre types, including SCFA. These are found to bind to G-protein coupled receptors on the surface of L-cells (Cani et al., 2013), increasing satiety and decreasing food intake by triggering gut hormone secretion (Cani et al., 2004; Delzenne et al., 2005). In fact, in these studies increases in GLP-1 and GLP-2 were associated with an improvement in host phenotype (ie. decreased hunger, increased satiety, decreased fat mass and body weight and improved diabetes) (Cani et al., 2004, 2005a, 2005b, 2007a, 2007b; Kok et al., 1998). This association was confirmed when only a partial improvement of the phenotype was seen after prebiotic treatment in GLP-1 and GLP-2 receptor knockout mice (Cani et al., 2006, 2009; Delzenne et al., 2007). The mechanism behind this is unknown, but it is thought to be dependent on an increased number of L-cells in the colon, indeed recorded in rodents after prebiotic treatment (Cani et al., 2007a; Everard et al., 2011; Wichmann et al., 2013). The increase in the number of these cells may be associated with a higher differentiation of stem cells, which is hypothesised to be influenced by prebiotic consumption (Clevers and Batlle, 2013). Whilst theoretical at present, an understanding of how L-cell numbers can be increased could also aid the treatment of type 2 diabetes patients who currently receive GLP-1 injections as their endogenous production is low.

In addition to describing the impact of the fermentation of carbohydrates, Professor Cani also presented data suggesting that protein fermentation influences the balance of gut peptides. Protein fermentation can lead to indole production also suggested to be able to modulate the production of GLP-1 in that very high amounts can block GLP-1 secretion, whereas lower amounts, in combination with ions such as calcium, can stimulate GLP-1 secretion (Chimerel et al., 2014).

Other studies suggest that SCFA do not only act on the gut but can directly reach the brain and modulate the balance of signals to influence appetite. An elegant study showed that labelled acetate, infused intravenously and colonically, could cross the blood–brain-barrier in mice (Frost et al., 2014).

Professor Cani stressed that the impact of fibre and prebiotic consumption on host energy balance has not only been seen in rodents. Human studies also reported moderate reductions in body weight (2–2.5 kg) and visceral fat mass in overweight or obese subjects consuming 15–30 g daily of prebiotics such as inulin or resistant starch for 6–24 weeks. These studies also showed reduced hunger, increased satiety, increased PYY, decreased glycaemia and decreased plasma LPS (Chambers et al., 2015b; Daud et al., 2014; Dehghan et al., 2014; Dewulf et al., 2013; Gibson et al., 2017; Lecerf et al., 2012; Morel et al., 2015; Parnell and Reimer, 2009; Pourghassem Gargari et al., 2013).

Besides bacterial SCFAs and indole, neurotransmitters produced by bacteria also constitute a possible mechanism underlying prebiotic effects (Cani and Knauf, 2016). The endocannabinoid system influences a wide range of biological functions including food intake, adipose tissue browning and inflammation (Cani et al., 2014). Professor Cani’s group has investigated whether specific gut bacteria can modulate the intestinal endocannabinoid system, focusing on the potential role of *Akkermansia muciniphila* in adipose tissue browning (via gut peptides and endocannabinoids) (Geurts et al., 2015; Muccioli et al., 2010). Studies of *A. muciniphila* treatment showed that this significantly increases the intestinal levels of two endocannabinoids found to be anti-inflammatory and one involved in the secretion of GLP-1 (Everard et al., 2013). Moreover, a strong correlation was found between *A. muciniphila* and L-cell numbers (Everard et al., 2011). Since this microbe is a propionate producer, it is also hypothesised to stimulate the production of GLP-1, GLP-2 and PYY.

In summary, various human and animal studies demonstrate that gut microbes have a considerable impact on host metabolism. This community can either act locally in the gut or can communicate with peripheral organs. Growing evidence suggest that metabolic products from the gut influence a myriad of metabolic pathways, contributing, among others, to the modulation of neurotransmitter production and hormonal balance (Rastelli et al., 2018).

## The role of microbiota in the prevention and treatment of cancer

Professor Julian Marchesi (Imperial College London and Cardiff University, UK) pointed out that it is not enough to consider only the genome and the epigenome of an individual to understand non-communicable diseases. Considering the microbiota is equally important given their functions and interactions with the host via metabolites (eg. SCFA, branched chain and decarboxylated amino acids, bile molecules) and proteins (Holmes et al., 2012). Our biology is so intertwined with microorganisms that our cells have receptors specifically for microbial metabolites. Furthermore, the composition of the gut microbiota can dictate how the host responds to drugs. Examples have shown that side effects related to a common colon cancer drug are caused by a gut bacterial enzyme (Wallace et al., 2010) and, conversely, that probiotics can interact with the immune system to promote the success of cancer treatments (Zitvogel et al., 2017).

The manifestation of several chronic diseases, including cancer, have an association with the microbiota found in the gut or other body sites (Nicholson et al., 2012). The gut microbiota can be the cause of an initial mutation as the result of an amensalistic relationship, defined as “an interaction in which one organism inflicts harm to another organism without receiving any costs or benefits” (Mougi, 2016). For example, colibactin, produced by certain strains of *E. coli* (a normal human commensal) and hydrogen sulphide produced by many gut bacteria, are both by-products of their normal bacterial metabolism, but at the same time are genotoxic molecules that can cause inflammation and trigger tumorigenesis. As these substances are part of their metabolism, they do not lend themselves to targeted preventative solutions for colorectal cancer (CRC) (Louis et al., 2014).

The vast variability in gut microbial composition clouds the issue of what constitutes a healthy versus an unhealthy microbiota. As such, the term “dysbiosis” may not be helpful as this also cannot be defined in concrete terms nor scientifically quantified (Olesen and Alm, 2016; Brüssow, 2020). Moreover, while a gut microbiota could be considered “normal” or “healthy” in one country, it could be different in another (De Filippo et al., 2010). Professor Marchesi suggests not to use the term “dysbiosis” indiscriminately, but only in those situations where bacteria appear in niches in which they are normally not found (eg. small intestinal organisms migrating to the large intestine after bariatric surgery).

In CRC, research seems to suggest that a specific “cancer microbiota” exists with specific communities living “on” or “off” the tumour. This work led to the development of the “driver/passenger hypothesis”, which suggests that toxin-producing (but not pathogenic) bacteria can drive the development of CRC. Once a carcinoma has established, the bacterial community will change, normally becoming less diverse, although with the same bacterial load. Only certain bacteria can thrive in the tumour environment and these are thought of as “passengers” (Tjalsma et al., 2012). In particular, *Fusobacterium* is present in far higher quantities on advanced tumours (Castellarin et al., 2012; Kostic et al., 2012; Marchesi et al., 2011). Furthermore, in a study on bacterial abundance and co-occurrence, the bacteria present appear to change along with the disease stage from a benign cluster to a pathobiont cluster. Interestingly, as tumours become more invasive this seems to revert (Marchesi et al., 2011). The association of specific mutations driving the tumour with specific bacteria has also been analysed. Ascertaining which bacteria are typically present on tumours could be useful in developing more specific testing for CRC, as metabolites characteristically produced by these species could be measured.

In terms of CRC treatment, Professor Marchesi proposes a “TIMER mechanism” (standing for translocation, immunomodulation, metabolism, enzymatic degradation and reduced diversity) in that the microbiota modulates the responses to chemotherapy at different stages in the oncology pathway, something that can be externally exploited for the benefit of treatment (Alexander et al., 2017).

Professor Marchesi’s group is interested in the proteome, particularly in proteases produced by gut bacteria, which are potential virulence factors in CRC and IBD (Steck et al., 2011). For example, in a cohort of >500 IBD patients, faecal protease activity was found to be high compared to healthy controls, likely to be metalloproteases driven by members of the Firmicutes (eg. clostridia, enterococci, lactobacilli). This correlated with high gut permeability and could be responsible for flare-ups in this patient group (Osborne and Marchesi, 2018).

In conclusion, newer ecologic concepts such as amensalism could help to guide us to a better understanding of the role of the microbiota in non-communicable diseases and the application of multimodal “omic” tools is required to determine the roles of the gut bacteria in cancer. Importantly, the microbiome needs to be considered in relation to cancer drug efficacy.

## Modelling the gut microbiota *in vitro* to assess food intervention efficacy and mode of action

Professor Tom Van de Wiele (Ghent University, Belgium) focused further on the usefulness of *in vitro* models. He began his talk by highlighting the fact that there is high interindividual variability in studies measuring the possible health effects of food ingredients. This variation could be explained by numerous factors influencing the individual composition of the microbiota, such as the mode of delivery, being breast or formula fed, diet, lifestyle choices or antibiotic use (Bolca et al., 2013). Due to this complex interindividual variation, it may not be helpful to study the average response from a group of subjects when investigating the impact of a specific food component.

Thanks to initiatives such as the Human Microbiome Project Consortium (Human Microbiome Project Consortium, 2012), knowledge relating to the impact of microbes on health and disease has expanded. Researchers have moved from focusing on the phylogenetic compositional differences of the microbiota to exploring their metabolic potential and further relationship to the host. Based on these findings, molecular and physical interactions between the microbiota and the host seem to be more important for homeostasis than microbiota functionality, which is fairly stable between individuals. However, some health effects may actually depend on the unique metabolic potency of a specific microbial group or species. A good example of this is the conversion of daidzein, an isoflavone present in soy, to equol by gut bacteria present in only 33% of Caucasians. An equol producing phenotype has been found to have an impact on vasomotoric symptoms (such as hot flushes), osteoporosis (bone mineral density) and CVD risk markers (LDL cholesterol and C-reactive protein) (Jackson et al., 2011). Gut microbial composition therefore dictates whether an individual will gain health benefits from consuming soy isoflavones or not.

Understanding interindividual differences also requires consideration of the different microenvironments that exist within the individual gastrointestinal tract (GIT), eg. the proximal and the distal colon, as well as the luminal and mucosal regions, offering different nutrient platforms to the microbiota (Bäckhed et al., 2005). Studies limited to the analysis of stool samples only may not represent all of these microbial environments. For this reason, Professor van de Wiele has dedicated part of his work to optimising the Simulator of the Human Intestinal Microbial Ecosystem (SHIME) model to mimic the individual intestinal environment as closely as possible. SHIME is an *in vitro* system that includes the stomach, small intestine, proximal and distal colon (Marzorati and Van de Wiele, 2016) and includes mucosal platforms through the inclusion of microcosms that are coated with mucins to which bacteria can adhere. This allows researchers to focus on luminal microbes and those that adhere to the mucus platform (Marzorati et al., 2014; Van den Abbeele et al., 2013). SHIME suspensions can also be cocultured with host cells such as epithelial cells, hepatocytes and immune cells to study the interactions between the microbiota, the gut barrier and the immune system.

Two studies were presented in which SHIME was used to investigate hops and wheat bran as important ingredients to support host health. Hops contain bioactives such as isoxanthohumol (IX) which is being investigated as a potential cancer drug and thought to be the most potent plant hormone. IX can be converted to 8-prenylnaringin (8-PN) which is structurally quite similar to oestradiol and has therefore been proposed for the treatment or prevention of oestrogen-related pathologies such as breast cancer and menopausal symptoms. The conversion of IX to 8-PN has been noted to vary between female individuals, ranging from 0 to 100% (Possemiers et al., 2006). When faecal microbiota from high, medium and low 8-PN producers were added to the SHIME model, the conversion phenotype was found to be preserved (Molly et al., 1994; Possemiers et al., 2006) and revealed that conversion primarily took place in the distal colon. Furthermore, it was found that *Eubacterium limosum*, an IX converting microbe producing 8-PN, could improve the 8-PN producing phenotype in both *in vitro* and animal models.

Fibre is another important ingredient that can support human health. Modern diets tend to lack sufficient fibre. This may be detrimental, as fibre provides fuel for our microbiota (Scientific Advisory Committee on Nutrition, 2015). Wheat kernels contain several layers including the pericarp and the aleurone layers which differ as to how easily fermentable they are by the gut microbes. Interestingly, research suggests that wheat bran can drive microbiota niche diversification (De Paepe et al., 2017). Microscopy imaging of bran fractions incubated in the SHIME model showed that gut microbes can colonise these fractions. Compositional analyses showed that at the genus level, wheat bran colonising groups were highly variable among individuals but *Clostridium* cluster XIVa was enriched in all of them. *Clostridium* cluster XIVa are known butyrate producers often present in the mucosal environment which have important roles in maintaining barrier function and in stimulating regulatory T cells (Atarashi et al., 2011) and are found in lower abundance in IBD.

Accumulating research is focusing on the gut bacterium *A. muciniphila* (Derrien et al., 2017). In mice, high fat diets supplemented with *A. muciniphila* (alive, pasteurised or with a purified membrane protein isolated from the strain) resulted in lower body weight, lower levels of circulating LPS and lower insulin resistance, but with a stronger gut barrier function and an increased number of goblet cells, as compared to a normal diet (Plovier et al., 2017). In the human gut, abundance of *A. muciniphila* is highly variable (Derrien et al., 2008). In an attempt to understand in depth the driving factors for *A. muciniphila* to colonise the gut, the impact of mucin on intestinal colonisation was investigated using the SHIME model. In that study, the colonic environment of eight individuals was compared and the presence of mucin appeared to be the most important factor, not only modulating *A. muciniphila* colonisation, but also driving metabolic networks of the background microbiota. This primarily relates to mucin being a host glycan which can also be used as an energy source for the microbiota and thereby impacting host microbe interactions in general.

In conclusion, *in vitro* simulations of the GIT, such as the SHIME model, allow for the replication of the interindividual variability and functional signature of the microbiota from different individuals. This is important when investigating a food component which can have a different stimulatory effect on the compositional and metabolic networks of the gut microbial community.

## Mechanisms of probiotic activities: the needle in the haystack or too many to understand?

Professor Sarah Lebeer (University of Antwerp, Belgium) started her presentation by pointing out that despite mechanistic studies on molecular interactions of probiotics being available in the literature, few examples exist where such molecular activities, measured by, eg. mutagenesis, microbiome sequencing or biomarker improvement, can adequately explain the targeted clinical effects or allow the prediction or selection of strains for particular clinical applications.

Professor Lebeer agrees with describing probiotics as “bags of functions” and categorised these numerous functions or molecular mechanisms of action into the following (Lebeer et al., 2018): (i) modulation of the composition and activity of the indigenous microbiota, at least temporarily; (ii) enhancement of epithelial barrier function; (iii) modulation of the immune system; (iv) modulation of systemic metabolic responses and (v) signalling via the central nervous system.

Individual probiotic microorganisms have strain-specific properties, but different strains belonging to a taxonomic group may share core functions. For example, when considering lactic acid bacteria, a core property is that they all produce lactic acid; gram positive probiotic strains all have TLR2 ligands and bifidobacteria all contain mucus-binding proteins (Sanders et al., 2018). From a molecular perspective, behind the “bags of functions” are intracellular effector molecules in the form of enzymes (eg. lactase, bile salt hydrolases) and metabolites (lactate, SCFA, amino acids and derivatives), as well as effector molecules on the cell wall such as pili (Lebeer et al., 2018). The combination of core and specific mechanisms defines the final effect of a specific probiotic.

*Lactobacillus rhamnosus* GG (LGG) is arguably one of the most studied probiotics. It possesses several effectors against pathogens, including lactic acid (Makras et al., 2006; van den Broek et al., 2018) and lectin-like molecules (Petrova et al., 2016) that act in the gut and other niches like the upper respiratory tract. Bacteriocins, autoinducer-2 and peptide-based quorum sensing were also suspected to be involved, but this remains to be further substantiated (Lebeer et al., 2007). There is, however, increasing evidence that LGG can actively modulate the microbiota (Durack et al., 2018; Harata et al., 2017; Korpela et al., 2016). Exactly how this occurs has not been fully explained, but various ways in which LGG interacts with host epithelial cells and the important effector molecules have been documented. These include pili, extracellular polysaccharides, lipoteichoic acid, secreted proteins such as Msp1 and Msp2, and CpG DNA which is involved in TLR-9 interactions (Segers and Lebeer, 2014).

Comparative genomics is an interesting approach to find conserved (probiotic) features beyond LGG. Within the *Lactobacillus casei* group, three separate phylogenetic clades (groups of organisms with a common ancestor) exist, comprising the species *L. paracasei*, *L. casei* and *L. rhamnosus* respectively (Wuyts et al., 2017). It has long been known that LGG is more adherent than most lactobacilli and this is thought to be due to the specific type of pili on their surface (Tuomola et al., 1999). SpaCBA pili are made up of three subunits and promote adhesion to host cells through a “zipper-like” mechanism (Tripathi et al., 2013). The presence of SpaCBA pili is limited to the genomes of *L. rhamnosus* and *L. paracasei* (Douillard et al., 2013), while the *L. casei* group appears to harbour fimbriae composed of serine-rich glycoproteins (Wuyts et al., 2017). Another clade-specific property is resistance to oxidative stress, thought to be involved in the mechanism of action of probiotics, eg. against IBD (Tomusiak-Plebanek et al., 2018). All *L. paracasei* genomes have superoxidase dismutase, while *L. casei* genomes have catalase (Wuyts et al., 2017).

A better understanding of how probiotics work in the GIT can also be extended to the prediction of activities and functions in other niches (ie. ear, nose and throat; skin and vagina). Probiotics should be able to adapt to the local stress conditions and available nutrients in the specific niche(s) of interest. In addition, to promote health effects, they must show antipathogenic effects, epithelial barrier protection or immunomodulation (Lebeer et al., 2008). For example, LGG has been shown to be able to inhibit urogenital *Candida* infections by preventing the formation of hyphae, at least in part due to its exopolysaccharides (Wuyts et al., 2017) and this is now being studied in a clinical trial upon application in a specifically formulated topical gel. Present work is looking at the potential role of LGG in the prevention of upper respiratory tract infections (URTIs).

Professor Lebeer concluded that while looking for effector molecules of probiotics can seem like looking for a needle in a haystack and mechanistic work is slow and time consuming, it is worthwhile, especially because certain molecules have more of an effect than others and are therefore more important to focus on as they help to explain probiotic mechanisms of action.

## Studies with *Lactobacillus casei* Shirota: what do they tell us?

### Immunological effects of probiotics

Dr Masanobu Nanno (Yakult Central Institute, Japan) explained the role of the gut microbiota in the development and maturation of the immune system during infancy. Perturbations of the microbiota early in life, if persistent rather than transient, can influence susceptibility to atopic, immune-mediated, metabolic and potentially neoplastic diseases in later life (Zeissig and Blumberg, 2014). Furthermore, gut bacterial composition and function can be altered at any life stage by various factors, including diet, severe mental stress, ageing, medication use and infection (Odamaki et al., 2016). These disturbances, termed dysbiosis, are associated with the occurrence of various diseases including diarrhoea, allergy, IBS, IBD or cancer, though cause and effect relationships have not always been established. In addition, the variation of gut microbial composition in diseased subjects is not uniform (Tsuji et al., 2018). In the future it may be possible to pinpoint exactly which compositional changes are critical in relation to disease (Nomoto and Matsuda, 2015).

Probiotics, defined as “*live microorganisms that*, *when administered in adequate amounts, confer a health benefit on the host*” (Hill et al., 2014; Joint FAO/WHO Working Group on Drafting Guidelines for the Evaluation of Probiotics in Food, 2007), may be promising agents for recovering from a disturbed microbiota or preventing the development of disease if consumed regularly as part of the diet. Safety, taxonomy and confirmation of health benefits are all important aspects of probiotic classification. The probiotic strain *L. casei* Shirota has been used by the food industry in Japan for more than 80 years. Dr Nanno presented data from several studies to support that *L. casei* Shirota survives the GIT, increases beneficial bacteria (lactobacilli and bifidobacteria) and SCFA in the gut and decreases harmful bacteria (such as *Clostridium perfringens*, *Staphylococcus* and *Pseudomonas*) and substances (such as *p*-cresol) (Bian et al., 2011; De Preter et al., 2004). Moreover, positive effects of *L. casei* Shirota have been shown on bowel habits and in reducing gut discomfort in the elderly (Koebnick et al., 2003; Sekita et al., 2015; Van den Nieuwboer et al., 2015). Findings were also shared from clinical studies showing the favourable effect of *L. casei* Shirota on infectious diarrhoea in children (Sur et al., 2011), antibiotic-associated diarrhoea (Pirker et al., 2013; Wong et al., 2014; Wright et al., 2015), upper respiratory tract infections (Fujita et al., 2013; Nagata et al., 2016; Van Puyenbroeck et al., 2012) and hypertension (Aoyagi et al., 2017).

One of the mechanisms by which probiotics exert their beneficial effects on humans is through modulation of the immune system. Researchers at the Yakult Central Institute are interested in how *L. casei* Shirota may have effects on the innate immune system measured by looking at natural killer (NK) cell activity. Low NK cell activity has been associated with a higher risk of cancer development (Imai et al., 2000) and *L. casei* Shirota consumption has been associated with a reduced incidence of superficial bladder and breast cancer in case–control studies (Aso et al., 1995; Toi et al., 2013), as well as a reduced reoccurrence of atypia of CRC cells in an intervention study (Ishikawa et al., 2005). Although the data looking at NK cell activity are not consistent, they point towards the existence of responders and non-responders (Dong et al., 2013; Nagao et al., 2000; Reale et al., 2012; Seifert et al., 2011; Shida et al., 2017; Spanhaak et al., 1998; Takeda et al., 2006). Dr Nanno speculated that *L. casei* Shirota has the potential to increase NK cell activity if it is consumed for a long enough period. *In vitro* studies have shown that interaction between activated monocytes and NK cells is important to enhance NK cell activity (Walzer et al., 2005) and experiments using human peripheral blood mononuclear cells indicate that *L. casei* Shirota triggers monocytes to produce IL-12 increasing the cytotoxicity of NK cells (Takeda et al., 2006). Furthermore, the cell wall polysaccharides of *L. casei* Shirota are thought to play a key role in the immune modulating activity, such as regulation of cytokine production (Yasuda et al., 2008). Type-1 polysaccharides (PS1) and type-2 polysaccharides (PS2) differ in that PS1 have a high molecular mass and a linear structure whereas the PS2 are smaller and have a branched structure (Nagaoka et al., 1990). When incubated with macrophages, mutants of *L. casei* Shirota that lack PS1 induced excessive production of proinflammatory cytokines compared to the wild-type probiotic, indicating that PS1 regulates the balance of cytokine production (Yasuda et al., 2008). Interestingly, in a colitis-associated cancer model in mice, the PS1-negative mutant strain did not modify the pathogenesis of dextran sulfate sodium-induced colon cancer, unlike the wild-type strain which prevented its development in association with the decreased production of the inflammatory cytokine IL-6 in the colonic mucosa (Matsumoto et al., 2009), further indicating that these polysaccharides are involved in the immune modulatory effect of *L. casei* Shirota. Additional data from *in vitro* work carried out using mouse peritoneal macrophages suggest that the cytokine production triggered by *L. casei* Shirota is not always consistent, as cytokine profiles appear to change depending on the presence or absence of teichoic acid derived from the cell wall of *L. plantarum* (Kaji et al., 2010). Dr Nanno ended his talk by hypothesising that, as *L. casei* Shirota can induce differing immune responses, depending on surrounding conditions (eg. presence or absence of inflammation), *L. casei* Shirota has the potential to have multiple effects, though more studies are required to fully understand how opposing effects can be exerted by the same strain and to define the right conditions for the application of the strain.

### Management of stress through the gut

Professor Kensei Nishida (The University of Tokushima Graduate School, Japan) shared data from clinical trials conducted to explore whether probiotics, such as *L. casei* Shirota, can relieve the stress-response in healthy medical students, related to taking an academic examination.

The existence of a bidirectional connection between the gut and the brain and its influence on our health has been known for a long time (Mayer, 2011). The gut–brain axis includes the central, the autonomic and the enteric nervous systems, as well as the hypothalamic-pituitary-adrenal (HPA) axis. Its function can be explained as hierarchically organised reflex loops, including the lowest reflex circuits contained within the enteric nervous system to the highest reflex loop involving areas of the brain that control emotional regulation (Mayer, 2011). Animal studies have indicated that the commensal microbiota can affect the development of the HPA stress response (Sudo et al., 2004) and it is now widely accepted that the enteric microbiota is part of this axis.

Stress, particularly chronic stress, is known to have a negative impact on wellbeing, including causing *physical* symptoms, such as abdominal dysfunction, as well as *psychological* symptoms, such as sleep disturbance and even depression. Professor Nishida and colleagues hypothesised that improvement of the gut environment using probiotics may have the potential to modulate the stress response and relieve stress-related symptoms. To investigate this, they pooled the results from three double-blind randomised, placebo-controlled trials that were conducted to examine the effects of *L. casei* Shirota on psychological and physiological stress responses in healthy medical students under academic examination stress (Takada et al., 2016). In these studies, the participants consumed either a fermented milk drink containing *L. casei* Shirota (1.0 × 10^11^ colony forming units/day) or a placebo drink daily for 8 weeks until the day before the academic examination. Outcomes measured included laboratory parameters (salivary cortisol), physical symptoms (abdominal and cold symptoms), psychological parameters (current mental health, transitory emotional stress experienced before an event), as well as anxiety as a personality trait. In these pooled analyses, the *L. casei* Shirota intervention inhibited the appearance of stress-related physical symptoms and significantly suppressed salivary cortisol levels when compared to the placebo group. However, anxiety scores increased gradually, peaking the day before the examination and did not differ between the intervention groups. Gene expression profiles were assessed using DNA microarrays. Interestingly, *L. casei* Shirota administration almost completely prevented additional changes in the expression of stress-response genes one day before the examination (Kato-Kataoka et al., 2016). The composition of the gut microbiota was also measured using 16S rRNA gene amplicon sequence analysis, and although there were no significant differences in the composition of phyla between the two groups at each time point, the proportion of Bacteroidetes tended to increase in the placebo group while it did not change in the probiotic group. *L. casei* Shirota administration was also able to preserve the phylogenetic diversity index (an indicator of alpha-diversity) before the examination, in contrast to placebo (Kato-Kataoka et al., 2016).

In a further double-blind, placebo-controlled trial following a similar protocol, Professor Nishida and colleagues aimed to evaluate whether probiotics could improve sleep quality or relieve stress-induced sleep disturbances in those students with an upcoming examination (Takada et al., 2017). In this trial, a total of 46 and 48 subjects consumed *L. casei* Shirota or placebo daily, 8 weeks prior to and 3 weeks after the examination. The absence of sleep disorders among the participants was ensured using the Pittsburgh Sleep Quality Index. Outcomes measured included subjective sleep quality, using the Oguri–Shiraka–Azumi (OSA) sleep inventory which evaluates five factors (sleepiness on rising, initiation and maintenance of sleep, frequent dreaming, recovery from fatigue and sleep length), anxiety levels using the State-Trait Anxiety Index (STAI) and overnight electroencephalography (EEG) recordings. The STAI anxiety scores of both groups increased significantly 2 weeks before the exam and did not show difference between the treatment groups. The STAI score peak coincided with the lowest OSA score, indicating that stress affected sleep. Total OSA scores did not differ between the two groups across the study period, but compared to placebo, probiotic treatment was associated with significant positive effects on scores for “sleepiness on rising” and “sleep length” (no significant effects on other OSA parameters were found), and on “delta power” (an index of sleep intensity measured by EEG) as the exam approached. Moreover, *L. casei* Shirota was also associated with preventing EEG changes that were seen in the placebo group, in whom sleep latency lengthened and percentage of deep sleep (stage 3 non-REM) decreased as the examination approached. These data suggest that probiotics, such as *L. casei* Shirota, may have a beneficial effect on sleep quality during a stressful period.

Professor Nishida concluded his presentation by speculating that the mechanism behind *L. casei* Shirota acting on the brain to prevent the onset of stress-induced symptoms, may be through stimulation of the afferent vagus nerve that modulates the reactivity of both the HPA axis and the sympathetic nerve, therefore controlling the cortisol response initiated by stress (Takada et al., 2016), although these theories are yet to be further substantiated by human and mechanistic studies.

### A double-blind, placebo-controlled, randomised trial of probiotic therapy *Lactobacillus casei* Shirota in stable cirrhotic patients

In liver cirrhosis, a pathological gut microbiota and bacterial translocation has been linked with immune dysfunction (Macnaughtan and Jalan, 2015). A pilot study of the probiotic strain *L. casei* Shirota in alcoholic cirrhosis demonstrated a significant improvement in neutrophil function (Stadlbauer et al., 2008).

Dr Jane Macnaughtan (University College London, UK) presented the results of a double blind, placebo-controlled study that aimed to evaluate the efficacy of *L. casei* Shirota with regard to improvement of immune function and effect on sepsis rates in patients with cirrhosis (Macnaughtan et al., 2017). In this study, 92 patients with cirrhosis of all causes and Child-Pugh score below 10, were randomised to receive *L. casei* Shirota or placebo, three times daily for six months. Randomisation was stratified by alcoholic and non-alcoholic aetiology (*n* = 46 per group). The researchers measured neutrophil function, plasma cytokines, endotoxin, serum bacterial DNA positivity, intestinal permeability and urine metabolomic profiles at day 0, month 1 and month 6.

The results showed that *L. casei* Shirota was associated with a significant improvement in neutrophil phorbol myristate acetate-induced ROS generation in patients with abnormal neutrophil function but not in the total population. *L. casei* Shirota also significantly reduced plasma monocyte chemotactic protein-1 concentrations. In a subgroup analysis, *L. casei* Shirota significantly lowered plasma IL-1β in patients with alcoholic liver cirrhosis, whilst IL-17A and macrophage inflammatory protein-1β were significantly reduced in non-alcoholic cirrhotic patients compared with placebo. Clinical events (infection and decompensation) were low in both groups and this inevitably impacted on any potential efficacy signals from *L. casei* Shirota. No significant differences in intestinal permeability, markers of bacterial translocation or urinary metabolomic profile were observed between groups.

Dr Macnaughtan concluded that *L. casei* Shirota treatment in patients with early cirrhosis is safe and associated with an improvement in immune function that appears to occur independently of bacterial translocation and without any effect on the metabolomic profile.

## Phage and probiotics: friends or foes?

Professor Colin Hill (University College Cork, Ireland) started by reminding us that we live in a microbial world and that viruses massively outweigh bacteria in numbers.

Viruses that infect bacteria and Archaea are called bacteriophages (or phages) and most of them remain uncharacterised. Bacteria and phages are foes, although they coexist in three different scenarios. In a lytic infection, a phage attacks a bacterium and injects its nucleic acid that hijacks the cell, turning it into a phage-producing factory which then bursts to release new progeny. The multiplication factor can be as high as 1,000, a replication rate that can easily allow a bacterial culture to be overtaken. In a symbiotic relationship known as lysogeny, a phage integrates its genetic material into the bacterial genome in the form of a prophage which is copied each time the bacteria multiply. The prophage can induce the lytic cycle at any time. Finally, a bacterium can be phage-resistant through different mechanisms such as: blocking adsorption, preventing nuclear material entry, cleaving phage nucleic acid [eg. by restriction/modification or by the Clustered Regularly Interspaced Short Palindromic Repeats (CRISPR)-Cas system, discovered through study of a phage attack of *S. thermophilus* cultures (Barrangou et al., 2007)], impeding phage assembly or aborting phage infection systems.

Phages are found wherever bacteria live, including probiotics. Several potential interactions may occur when phages and probiotic bacteria meet. Phages can: (i) disrupt commercial probiotic production, (ii) kill probiotics entering the host, (iii) enter the lytic cycle *in vivo*, acting as markers for probiotic carriage; (iv) transfer genes to/from probiotic strains and (v) influence probiotic mechanisms of action.

Disruption of commercial probiotic production by phages is a persistent threat. Bacteriophages can cause extensive disruption to dairy fermentation due to their high virulence and their inhibitory effect on lactic acid formation. They can act against *Lactococcus*, *Lactobacillus*, *Leuconostoc* and *Streptococcus* species. Sophisticated methodologies have been developed in order to prevent phage infection during food production. For example, in cheese-making, closed systems are used along with phage-inhibiting media, rotation of starter cultures and direct inoculation. Yet, it has been discovered that phages appearing in “abnormal fermentations” had curiously occurred in closed tanks which should have been sterile. Indeed, these phages have been seen to come out of the probiotic strains themselves (Shimizu-Kadota et al., 1983). This points to, besides the implementation of closed systems, the possibility of using a strain resistant to specific phages. A successful example of this alternative was achieved by Shimizu-Kadota and colleagues who were able to develop a prophage-cured mutant which retained all the probiotic properties of a strain for making a fermented beverage (Shimizu-Kadota and Sakurai, 1982).

Bacterial genes may possess a CRISPR locus, representing a history of phage attack. A long history of exposure to phages has been demonstrated in *B. longum* as CRISPR loci are widespread (Hidalgo-Cantabrana et al., 2017). A study employing electron micrographs to study pili of LGG (which has at least three prophage regions), noted that isometric bacteriophages could be observed in the background, possibly indicating that the phage components encoded by one of the genome islands are functional, and that phages can therefore emerge during culture (Kankainen et al., 2009). Phage resistant mechanisms can be stacked, rotated or mixed by food manufacturers to overcome phage attacks, though there may be regulatory issues if more than one version of the same bacteria is being used within probiotic production.

Phages have the potential to kill probiotics entering the host. In fact, phages against *Lactobacillus paracasei* NFBC338 used to make a commercially available cheese, *L. rhamnosus* Lc 1/3 (related to LGG) and *E. coli* Nissle 1917 have been isolated from sewage and waste water (Alemayehu et al., 2009; Tuohimaa et al., 2006) indicating that phages against probiotics are present in the human body. Lysogenic phages can become lytic *in vivo* when facing a hostile environment, leading to the question of whether phages are present in the gut ‘waiting’ until individuals consume probiotics and whether this could explain observations of responders versus non-responders in clinical trials with probiotics. Furthermore, could constant consumption of one probiotic in high numbers select for an expansion of lytic phages? Investigating metagenomic databases for answers would be of interest.

Professor Hill also raised the question of whether phages can enter the lytic cycle *in vivo* and act as markers for probiotic carriage. Phages can be characterised within faeces using phageomics (viromics), a process consisting of physical separation of virus-like particles, DNA and RNA extraction and sequencing thereof by next generation sequencing methods and bioinformatic analysis (Shkoporov et al., 2018). Using this methodology to characterise samples from ten individuals, only a maximum of 0.5% of the phages found were known, indicating vast gaps in the current knowledge about them (Shkoporov et al., 2018). Phages are strain-specific, but for most of them the target is unknown. Nevertheless, such data suggest that they could be used as biomarkers for probiotic carriage. For example, phages differ between faecal samples from infants and elderly individuals in that more bifidobacterial phages (BifΦ) and lactococcal phages are present in infants. Moreover, phageomics can distinguish 12 month old infants by delivery mode (vaginal vs. caesarean section) based on BifΦ presence while bacterial 16S rRNA analysis cannot discriminate between these groups (McCann et al., 2018). There is also evidence that phages play a role in the modulation of bacterial communities in the infant gut (Lugli et al., 2016).

He continued explaining that experiments *in vitro* have shown that phages can induce and facilitate gene transfer between bacteria, including probiotic strains. This could be used to human advantage by transferring genes with therapeutic interest, eg. chemokine genes for the expression of anti-HIV molecules (Damelin et al., 2010).

The interference of phages with probiotic mechanisms of action was illustrated by a study in which a prophage-cured derivative of *S. thermophilus* was found to be less effective when competing with pathogenic bacteria (*Salmonella*, *Listeria* and *Staphylococcus aureus*) for adhesion on HT29 epithelial cells as compared to a prophage-containing strain (Guigas et al., 2016), emphasising the importance of checking if probiotic traits remain in cured strains.

Professor Hill concluded that bacteria and phages are neither friends nor foes, but they are “in a relationship” and this needs to be considered when producing probiotics or performing studies with them. Phages can be useful in protecting against infectious bacteria and their ability to transfer plasmid genes to probiotics (Baugher et al., 2014).

## Concluding remarks

The meeting was closed by Dr Bruno Pot (Yakult Europe, The Netherlands), whose concluding remarks highlighted the complexity of the probiotic–host relationship, therefore the difficulty of providing the scientific support needed for the substantiation of health claims for probiotics, and the fact that consumers are also now demanding, and are entitled to, information about the relationship between food products and health. Despite there being no health claims for probiotics accepted in Europe as yet, data presented at this meeting have demonstrated the many potential clinical applications for them.

## Notes on Contributors

Stacey Lockyer has worked as a nutrition scientist at the British Nutrition Foundation since 2015, having completed her MSc and PhD at the University of Reading. Marisol Aguirre has published articles in the field of gut health focused on a compositional and functional microbiota signature from obese individuals, the morphology of a healthy intestine in broilers and *in vitro* gut modelling. She is a member of the Science Department of Yakult Europe. Louise Durrant is a Registered Dietitian and Science Manager at Yakult UK Ltd. After completing her dietetic degree at the University of Surrey (2011), she remained at the University to conduct a PhD in Nutritional Sciences focusing on the comparative efficacy of the two forms of vitamin D (2016) before joining Yakult UK shortly thereafter.  Bruno Pot, PhD in microbiology from the University of Ghent, Belgium, has performed research in the area of lactic acid bacteria for close to 35 years. He is currently Science Director Europe at Yakult Europe (The Netherlands) and Guest Professor at the Vrije Universiteit Brussels (Belgium) and also involved in organisations as IPA, PRI, ILSI, LABIP, ISAPP. Kaori Suzuki MD, PhD has published several basic, translational, and clinical research articles on diverse biomedical areas. She works as Human Studies Manager at the Science Department of Yakult Europe.
